# Anti-Obesity Effects of *Petasites japonicus* (Meowi) Ethanol Extract on RAW 264.7 Macrophages and 3T3-L1 Adipocytes and Its Characterization of Polyphenolic Compounds

**DOI:** 10.3390/nu12051261

**Published:** 2020-04-29

**Authors:** Eun Mi Ahn, Gelila Asamenew, Heon Woong Kim, Sang Hoon Lee, Seon-Mi Yoo, Soo-Muk Cho, Youn-Soo Cha, Min-Sook Kang

**Affiliations:** 1 Department of Food Science and Human Nutrition, Jeonbuk National University, 567 Baekje-daero, Jeonju, Jeollabuk-do 54896, Korea; 2 Department of Agrofood Resources, Food and Nutrition Division, National Institute of Agricultural Sciences, 166 Nongsaengmyeong-ro Wanju-gun, Jeollabuk-do 55365, Korea

**Keywords:** *Petasites japonicus*, anti-adipogenic effects, anti-inflammation, anti-lipogenesis, characterization, ployphenolic compounds, flavonoids, UPLC-DAD-QToF-MS

## Abstract

Koreans have been consuming *Petasites Japonicus* (PJ) as food. Although the therapeutic effect of PJ on allergic or inflammatory reactions associated with asthma has been proven, its effect on obesity is unclear. Therefore, the present study was aimed to assess the obesity related anti-inflammatory and anti-adipogenic effects of ethanol extract PJ (EPJ) on the inflammatory response in RAW 264.7 macrophages and on differentiation in 3T3-L1 adipocytes. In addition, the polyphenolic compound was quantitatively characterized from the EPJ using ultra performance liquid chromatography coupled with diode array detector, quadrupole time-of-flight-mass spectrometry (UPLC-DAD-QToF-MS). In RAW 264.7 or 3T3-L1, reduction of nitric oxide (in macrophages) production as well as monocyte chemoattractant protein-1 and tumor necrosis factor-α were observed. Treatment of EPJ in adipocyte differentiation showed an improvement in adiponectin and lipid accumulation and a significant reduction of PPARγ and FABP-4 mRNA expression levels. On the other hand, mRNA expression of UCP-1, PPARα, and ACO increased in the EPJ treated group. In addition, a total of 26 polyphenolic compounds were detected and of which 12 are reported for the first time from PJ. The higher content of diverse polyphenolic compounds presented in EPJ might be responsible for the observed anti-inflammatory and anti-adipogenic effect. These results suggest that PJ is valuable in improving obesity-related inflammatory responses.

## 1. Introduction

Obesity is a state of low-grade, chronic inflammation and a major cause of insulin resistance that gives rise to type 2 diabetes [1,2]. Adipose tissue is an endocrine organ that secretes various types of cytokines, termed adipokines [3]. Macrophage infiltration of adipose tissue is triggered during the inflammatory response, and inflammatory cytokine secretion is increased by activated macrophages [4]. Inflammation arising from obesity increases nitric oxide (NO) causing metabolic disorders due to the increase in cytokines and reducing protective factors such as adiponectin [5]. Adiponectin is produced in adipose tissue [6] and was found to be decreased in obesity [7,8,9]. This downregulation has not been fully explained. Supplementation by differing forms of adiponectin was able to improve insulin control, blood glucose, and triglyceride levels in mouse models [10]. It has been demonstrated that monocyte chemoattractant protein 1 (MCP-1) and tumor necrosis factor-α (TNFα) are important pro-inflammatory biomarkers involved in the development of obesity induced meta-inflammation [6]. MCP-1 is a chemokine that mediates obesity-induced insulin resistance [11]. TNFα is a cytokine found to be elevated in obesity and decreased when adiponectin is involved in lipogenesis [12]. Obesity induced meta-inflammation leads to non-alcoholic fatty liver disease (NAFLD), lipid accumulation due to insulin resistance in skeletal muscle, atherosclerosis, and β-cell failure/death in the pancreas [4].

Peroxisome proliferator-activated-γ (PPARγ) is a major adipogenic factor and is also important for the maintenance of a fully differentiated state in culture and mice [13,14]. As insulin sensitizers, PPARγ agonist drugs known as thiazolidinediones (TZDs) have side effects, such as hepatotoxicity/liver failure or body fat increment [15]. In recent years, primary health care has prioritized disease prevention rather than medical treatment. Consequently, the importance of a healthy diet has been strongly emphasized. For this reason, extensive research has been carried out on the functionality of foods [16,17]. Polyphenols have various health functions. Polyphenols from fruit and vegetable sources have been found to improve weight management difficulties in diabetes, neurodegenerative disease, and cardiovascular disease [18,19]. 

*Petasites japonicus* (PJ, Korean Name: Meowi, English name: Butterbur) is a commonly consumed vegetable in Korea. It is usually consumed as soups or seasoned vegetable dishes, *Namul*. It is a perennial plant belonging to the Compositae family [20]. Research has found it to be effective as a natural remedy for asthma and allergic diseases [21,22]. In Europe, the root extract of the *Petasites* species is used to treat migraines and ulcers in addition to asthma prevention. It has also been reported to have excellent anticancer properties, cholesterol lowering effects, and oxidative stress reduction effects as free radical scavenger [23]. However, the anti-inflammatory effects of PJ on obesity and its related inflammatory responses remain unclear. In this study, the obesity related anti-inflammatory and anti-adipogenic action of ethanol extracted PJ (EPJ) in RAW 264.7 macrophages and 3T3-L1 adipocytes were evaluated. In addition, the EPJ were further quantitatively characterized for flavonoid and phenolic acids using ultra performance liquid chromatography coupled with diode array detector, quadrupole time-of-flight-mass spectrometry (UPLC-DAD-QToF-MS).

## 2. Materials and Methods

### 2.1. Sample Preparation and Reagents

Edible portions (leaves and stalk) of PJ samples were frozen at −70 °C; they were freeze-dried and powdered. EPJ was prepared by extracting the sample with 80% ethanol using the soxhlet extraction method, and the crude EPJ were freeze-dried. EPJ was dissolved in dimethyl sulfoxide (DMSO, Cat: D8418; Sigma-Aldrich, Saint Louis, MO, USA) and diluted in cell culture medium. The final concentration of DMSO was the same in all samples at 0.1%. Dulbecco’s Modified Eagle’s Medium (DMEM, Cat: D6429) was purchased from Sigma-Aldrich. Fetal bovine serum (FBS, Cat: 16000-044) and bovine calf serum (BCS, Cat: 16170-078) were purchased from the USA (Gibco-Invitrogen, Carlsbad, CA, USA).

Penicillin/streptomycin (Cat: P4083), lipopolysaccharides (Cat: L6529), Griess reagent and 3-(4, 5-dimethylthiazol-2-yl)-2, 5-diphenyltetrazolium bromide (MTT, Cat: M5655), 3-isobutyl-1-methylxanthine (IBMX, Cat: I5879), dexamethasone (DEX, Cat: D4902), and insulin (Cat: I5500) were obtained from Sigma-Aldrich. 

Caffeic acid, 4-*O*-caffeoylquinic acid, 5-*O*-caffeoylquinic acid, 3,4-di-*O*-caffeoylquinic acid, 3,5-di-*O*-caffeoylquinic acid, 4,5-di-*O*-caffeoylquinic acid, 3,4,5-tri-*O*-caffeoylquinic acid, galangin and 2,4,5-trimethoxycinnamic acid were purchased from Sigma Aldrich Co. (St. Louis, MO, USA). 1,5-di-*O*-caffeoylquinic acid was purchased from Avention (Incheon, Republic of Korea), 5-*O*-feruloylquinic acid from Chem Face (Wuhan, China), and 5-*O*-caffeoylquinic acid methyl ester was purchased from (Genay Cedex, France).

### 2.2. Cell Culture and Viability

The RAW 264.7 and 3T3-L1 cells were purchased from the Korea cell line bank (Seoul, Korea). RAW 264.7 macrophages were cultured in DMEM supplemented with 10% FBS, 100 U/mL of penicillin and 100 μg/mL of streptomycin at 37 °C in a humidified 5% CO_2_ atmosphere.

3T3-L1 adipocyte differentiation, as an obese model, was carried out as previously described [24,25,26]. Once cells reached confluence, the cells were incubated in the differentiation medium containing EPJ or troglitazone (positive control reagent) with MDI (methyl-isobutylxanthine 0.1 mM, dexamethasone 0.4 μM, insulin 10 μg/mL) medium for 72 h. On day 3 (D-0), medium was changed to insulin (5 μg/mL) with EPJ or troglitazone medium. The medium was renewed every 2 days for 10 days [22]. Measurement of cell viability was performed by MTT assay. RAW 264.7 macrophages and 3T3-L1adipocytes were seeded at approximately 1 × 10^5^ cells/200 μL/well and 2 × 10^3^ cells/200 μL/well, respectively, in 96 well plates and incubated for 2 h. The sample was dissolved in DMSO to a concentration of 2 g/mL and diluted in culture medium before use. The final concentration of DMSO for all experiments remained constant at 0.1%. After incubating for 18 h, 0.5 mg/mL MTT solution was added in a concentration of 20 μL to each well and incubated for another 2 h. After MTT removal, the colored formazan was dissolved in 200 μL of DMSO. The color intensity was measured at 550 nm using a microplate reader (Softmax pro; Molecular Devices, Sunnyvale, CA, USA). 

### 2.3. Measurement of Nitric Oxide

The RAW 264.7 macrophages were treated with diluted EPJ in Opti-mem (Gibco-Invitrogen; Cat: 31985) at different concentrations (0.01 and 0.1 mg/mL). At the same time, lipopolysaccharides (LPS) was added at a concentration of 100 ng/mL to make the obesity model. NO concentration in the culture medium was determined using the Griess reagent. The optical density (O.D.) was determined at 570 nm with a microplate reader (Softmax pro). NO values were corrected based on the cell viability. 

### 2.4. Oil-red O Staining

Oil-red O staining was performed as described previously [27]. Adherent cells were rinsed with phosphate buffer saline (PBS) and fixed with 10% formalin (neutral buffered) for 2 h, followed by rinsing with PBS. After rinsing, staining with Oil-red O was performed for 3 h. Plates were rinsed with distilled water (DW) and images of cells on the plates were taken. For quantification, the dye was extracted by DMSO, and absorbance was determined at 510 nm (Softmax pro). 

### 2.5. Measurement of Chemokine and Adipocytokines

Cytokines released from RAW 264.7 and 3T3-L1 were measured in their culture supernatants using the sandwich ELISA method. Analysis was performed with mouse TNFα (Cat: 555179), MCP-1(Cat: 555212) from BD Pharmingen (San Diego, CA, USA) and adiponectin (Cat: DY1119) from R&D system kits (Minneapolis, MN, USA). All data were analyzed as recommended by the manufacturer. 

### 2.6. Analysis of mRNA Expression

RNA purification from cultured cells was performed using an mRNA purification kit (Qiagen, Hilden, Germany, Cat: 74804;). The absorbance was measured using a spectrophotometer (Nanodrop 2000; Thermo Fisher Scientific, Wilmington, Delaware, USA). cDNA synthesis (Qiagen, Cat:205311) and quantitative polymerase chain reaction (qPCR) were performed using the QuantiTect SYBR Green PCR kit (Qiagen, Cat: 204143) and Step one plus Real time PCR System (Applied Biosystems, Foster City, CA, USA). Production of the following target genes and internal controls were assessed: TNFα, MCP-1, Adiponectin, PPARγ, fatty acid binding protein 4 (FABP-4), acetyl-coA carboxylase (ACC), uncoupling protein-1 (UCP-1), UCP-2, UCP-3, peroxisome proliferator-activated-α (PPARα), acyl CoA oxidase (ACO), carnitine palmytoyltransferase-1 (CPT-1), acidic ribosomal phosphoprotein P0 (36B4), and glyceraldehydes 3-phosphate dehydrogenase (GAPDH). The product of gene amplification was calculated by relative quantification. The ΔΔCT value method was used to measure relative quantification [28]. Quantitative reverse transcription polymerase chain reaction (RT-PCR) values were normalized to endogenous control genes, GAPDH or 36B4. The values were expressed as fold change over control. 

### 2.7. Extraction of Phenolic Acids and Flavonoids 

Crude extract was prepared by extracting 100 g of leaves and stalk part of PJ powder using 80% ethanol by soxhlet extraction method for 24 h. Then, the crude extract solvent was removed by rota evaporator and freeze-dried for complete drying of EPJ. Phenolic acid and flavonoid purification from the crude extract were conducted according to the method described by Kim et al. (2012) with minimum modification [29]. A total of 100 mg of EPJ powder was mixed with 10 mL of extraction solvent (methanol: water: formic acid = 80:15:5, *v*/*v*/*v*) containing 250 mg/L of 2,4,5-trimethoxycinnamic acid as the internal standard for phenolic acid analysis. The mixture was vortexed, stirred with an orbital shaker for 30 min at 200 rpm and centrifuged (LABOGENE 1580R, Korea) for 15 min at 3000 rpm, 10 °C. The supernatant was filtered using a syringe filter (0.45 μm, PVDF, Whatman, Kent, England). A total of 0.5 mL of the filtrate was diluted with water to 5 mL of final volume. The crude phenolic extract was then isolated by solid phase extraction method using Sep-Pak C18 cartridge (Waters Co., Milford, MA, USA). The cartridge was activated by washing with 2 mL of methanol, followed by 2 mL of water for conditioning. Then the diluted phenolic extract was loaded into the cartridge, and impurities were removed by washing with 2 mL of water. Finally, the crude phenolic acid was eluted from the cartridge using 3 mL of methanol. The purified extract was concentrated using N_2_ gas, and then re-dissolved with 0.2 mL of the extract solvents without internal standard prior to instrumental analysis. For flavonoids analysis, 100 mg of crude ethanol extract was taken and extracted with the solvent system (methanol: water: formic acid = 50:45:5, *v*/*v*/*v*) containing 20 μg/mL of galangin as an internal standard. The extraction and purification steps were followed as described above. All experimental analyses were performed in triplicates.

### 2.8. UPLC-DAD-QTOF/MS Conditions

Polyphenolic compound profiling was conducted by UPLC-DAD-QToF/MS (Waters Micromass, Manchester, UK). UV-visible spectra were recorded in the region from 210 to 400 nm. Separation was conducted using Cortexs^®^ UPLC^®^ T3 C18 column (2.1 mm × 150 mm, 1.6 µm) (Waters Co., Milford, MA, USA). Chromatographic conditions were set as column temperature 30 °C; injection volume, 1 μL; mobile phase consists of 0.1% formic acid in water (A) and 0.1% formic acid in acetonitrile (B). The gradient profile for phenolic acid analysis was as follows: 0–4 min, 2% B; 8 min, 4% B; 20 min, 7% B; 32 min, 11% B; 55 min, 15% B; 75–77 min, 25% B; 85–87 min, 50% B; 90–92 min, 90% B; 95–100 min, 2% B. Flow rate was 0.35 mL/min and the chromatograms analysis was carried out at 280 nm and 320 nm wavelengths. The gradient condition used for flavonoid was as follows: 0 min 5% (B), 20 min 25% (B), 25 min 50% (B), 30–32 min 90% (B), 35–40 min 5% (B). Flow rate was 0.30 mL/min and the chromatograms analysis was conducted at 280 nm and 350 nm wavelengths. The mass spectrometric settings used were: electrospray ionization source operating in positive mode (+ESI), capillary voltage was set to 3.5 kV, desolvation gas 1020 L/h, sample cone voltage 40 V. The source and desolvation temperatures were 140 and 500 °C, respectively. Data were recorded in the mass range of 50–1200 *m/z* in full scan mode.

### 2.9. Statistical Analysis

Data were expressed as mean ± standard deviation of four independent determinations. The statistical significance of any difference was determined by Student’s t-test. The SPSS 12.0 software package (SPSS, Chicago, IL, USA) was used for statistical analysis. A significant value was defined as * *p* < 0.05, ** *p* < 0.01, and *** *p* < 0.001.

## 3. Results

### 3.1. EPJ Inhibits NO Production in LPS-Stimulated Macrophages 

**Figure 1:**
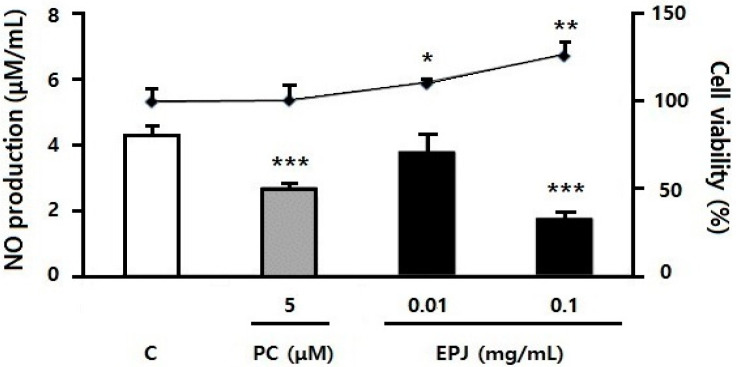
Extracted *Petasites Japonicus* (EPJ) inhibited lipopolysaccharides (LPS)-induced nitric oxide production in RAW 264.7 macrophages. All groups were incubated with LPS (100 nM) and positive control (PC, troglitazone 5 μM) or 0.01 or 0.1 mg/mL EPJ. The three treatment groups (PC and both of EPJ groups) were compared with the control (C; LPS treated only) group. Data are mean ± S.D. of four independent determinations. * *p* < 0.05, ** *p* < 0.01, *** *p* < 0.001.

Cell viability and NO production of RAW 264.7 macrophages are presented in Figure 1. NO values were corrected for cell viability. Cell viability increased significantly in 0.01 and 0.1 mg/mL of EPJ groups compared with the C group (LPS treated only). NO was significantly reduced in the PC group compared with the C group (*p* < 0.001, 38%). NO values were not statistically different in the EPJ 0.01 mg/mL group but were significantly reduced in the EPJ 0.1 mg/mL group compared with the C group (*p* < 0.001, 59%).

### 3.2. EPJ Inhibits MCP-1 and TNFα in LPS-Stimulated Macrophages

**Figure 2:**
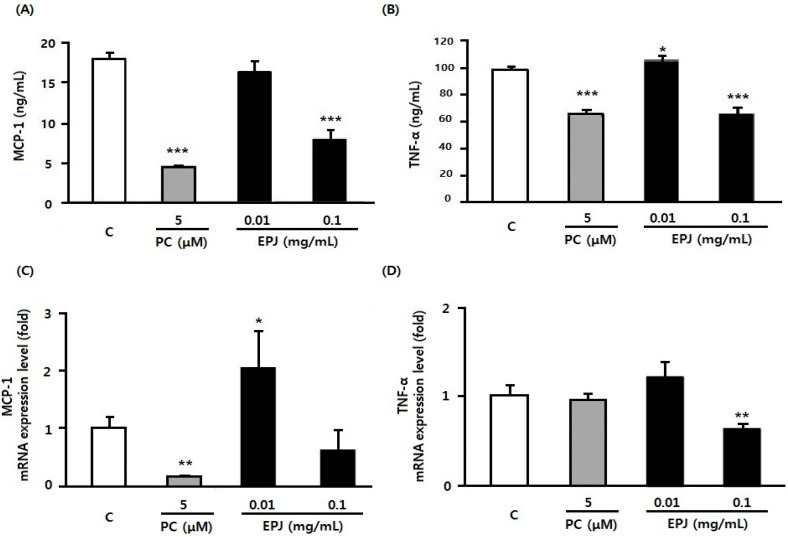
EPJ decreases the secretion and mRNA expression of MCP-1 and TNFα in LPS-stimulated RAW264.7 macrophages (**A**–**D**). Protein (**A**,**B**) and mRNA quantification (**C**,**D**) were performed. MCP-1 and TNFα expression were evaluated using glyceraldehydes 3-phosphate dehydrogenase (GAPDH) as the endogenous control gene. Troglitazone (5 μM) was used as the positive control. The three treatment groups (PC and both of EPJ groups) were compared with the control (**C**) group. Data are mean ± S.D. of four independent determinations. * *p* < 0.05, ** *p* < 0.01, *** *p* < 0.001.

Protein secretion and mRNA expression of MCP-1 and TNFα are presented in Figure 2A–D. MCP-1 and TNFα secretion levels were similar to the NO secretion patterns. Moreover, the PC and EPJ 0.1 mg/mL groups were significantly decreased compared to the C group (*p* < 0.001, Figure 2A,B). The mRNA expression of MCP-1 was significantly decreased in the PC group compared to the C group (Figure 2C, *p* < 0.01). Expression in the EPJ 0.01 mg/mL group was significantly increased. However, there were no significant differences in the EPJ 0.1 mg/mL group (Figure 2C). The mRNA expression of TNFα was significantly decreased in the EPJ 0.1 mg/mL group compared with the C group (Figure 2D, *p* < 0.01). There were no differences in the PC and EPJ 0.01 mg/mL groups compared with the C group. However, the protein production and mRNA expression of MCP-1 and TNFα showed generally similar patterns. Cytokines secretion (MCP-1 and TNFα) as inflammatory markers were found to be improved in the EPJ 0.1mg/mL group than the EPJ 0.01mg/mL group. 

### 3.3. EPJ Improves MCP-1 and Adiponectin in Differentiated 3T3-L1 Adipocytes

**Figure 3:**
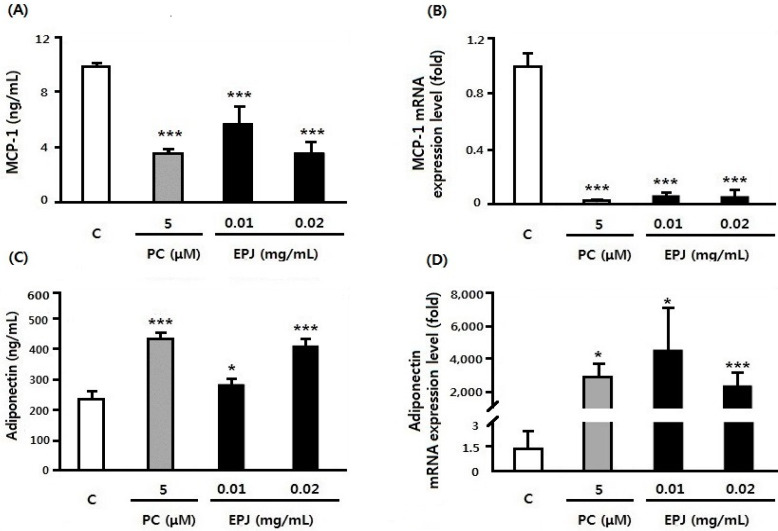
EPJ inhibited MCP-1 and increased adiponectin secretion in differentiated 3T3-L1 adipocytes. The qRT-PCR was performed to detect the expression of MCP-1, adiponectin using 36B4 as the endogenous control. The three treatment groups (PC and both of EPJ groups) were compared with the control (**C**) group. Data are mean ± S.D. of four independent determinations. (**A**) Concentration of MCP-1 (ng/mL). (**B**) mRNA expression level of MCP-1 (fold). (**C**) Concentration of adiponectin (ng/mL). (**D**) mRNA expression level of adiponectin (fold). * *p* < 0.05, ** *p* < 0.01, *** *p* < 0.001.

Protein and mRNA expression of MCP-1 and adiponectin in differentiated 3T3-L1 adipocytes following EPJ treatment is shown in Figure 3. MCP-1 protein levels were significantly decreased in the PC, EPJ 0.01, and 0.02 mg/mL groups compared with the C group (differentiated cells without PC or EPJ, *p* < 0.001, Figure 3A). Especially, EPJ 0.02 mg/mL showed a reduction effect similar to the PC group. MCP-1 protein levels were decreased by 64% in the PC and EPJ 0.02 mg/mL group compared with the C group. Also, the mRNA expression levels of MCP-1 were significantly decreased in the PC (98%), EPJ 0.01 mg/mL (94%), and 0.02 mg/mL (95%) groups compared with the C group (*p* < 0.001, Figure 3B).

Adiponectin protein levels were significantly increased in the PC (*p* < 0.001, 82%), EPJ 0.01 (*p* < 0.01, 19%), and 0.02 mg/mL (*p* < 0.001, 72%) groups compared with the C group (Figure 3C). Adiponectin mRNA expression levels were increased in the PC (*p* < 0.01, 2 × 10^5^%), EPJ 0.01 (*p* < 0.01, 3 × 10^5^%) and 0.02 mg/mL groups (*p* < 0.001, 1.6 × 10^3^%, Figure 3D). It could be confirmed that EPJ effectively reduced obesity related cytokine MCP-1 secretion and increased adiponectin [30].

### 3.4. EPJ Decreases Lipid Accumulation in Differentiated 3T3-L1 Adipocytes 

**Figure 4:**
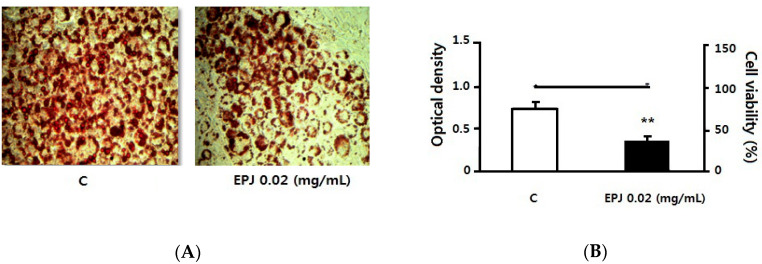
EPJ decreased lipid accumulation in methyl-isobutylxanthine (MDI) medium induced by 3T3-L1 adipocyte differentiation. (**A**) Oil red O image shows that lipid accumulation was visibly decreased in the EPJ 0.02 mg/mL group compared to the control (**C**) group. (**B**) Oil-red O staining was measured semi-quantitatively and optical density was significantly decreased in the EPJ 0.02 mg/mL group compared to the C group. Data are mean ± S.D. of four independent determinations. ** *p* < 0.01.

Figure 4 shows Oil red O staining with microscopic images of cells (Figure 4A) and was quantified by measuring the O.D. values (Figure 4B). In order to determine the treatment concentration, cell viability was first tested. EPJ did not affect cell viability at 0.005~0.02 mg/mL (data not shown). As shown in Figure 4A, lipid accumulation of adipose cell visibly and O.D. values (*p* < 0.01, 52%, Figure 4B) were significantly decreased in the EPJ 0.02 mg/mL group compared to the C group.

### 3.5. Anti-Lipogenic Effect of EPJ in Differentiated 3T3-L1 Adipocytes 

**Figure 5:**
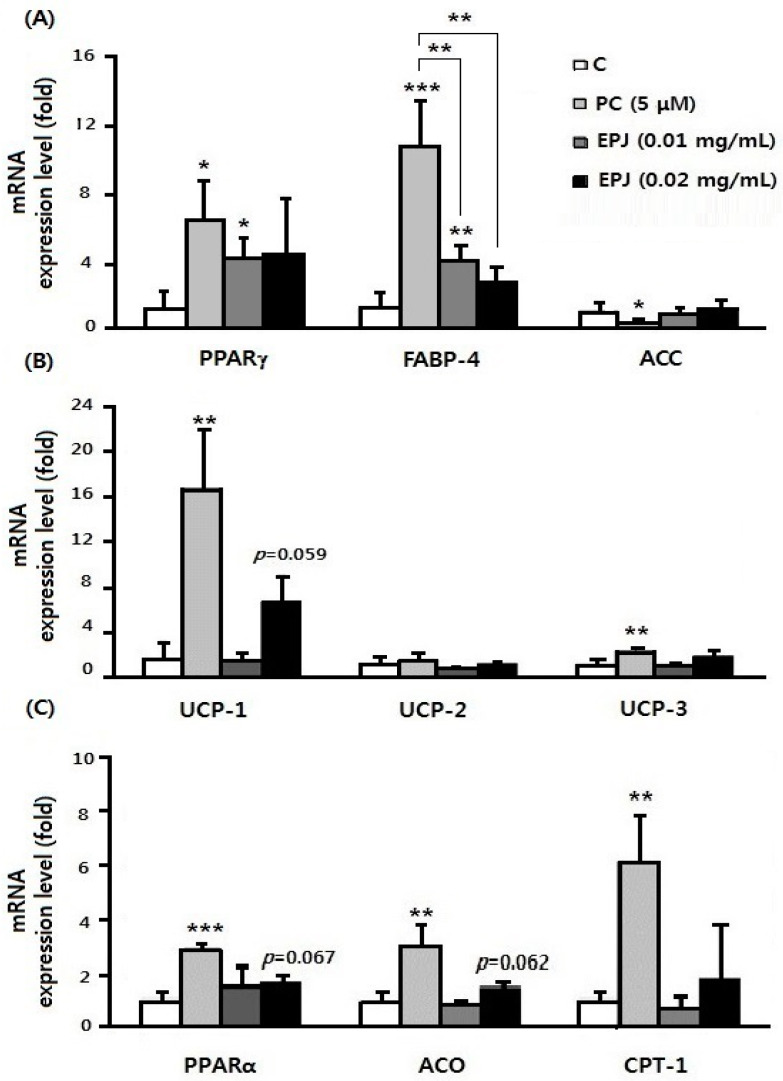
In 3T3-L1 adipocytes, qRT-PCR was performed to detect the expression of PPARγ, FABP-4, ACC, UCP-1, UCP-2, UCP-3, ACO, CPT-1, and PPARα (**A**–**C**) using 36B4 as the endogenous control. The three treatment groups (PC and both of EPJ groups) were compared with the control (**C**) group. Data are mean ± S.D. of four independent determinations. * *p* < 0.05, ** *p* < 0.01, *** *p* < 0.001.

In Figure 5, C is differentiated adipocytes without any treated and the other three groups are treated with PC or EPJ to differentiated adipocytes. The mRNA expression level of adipogenic gene PPARγ was significantly increased in the PC and EPJ 0.01 mg/mL groups (*p* < 0.05, Figure 5A). The PPARγ target gene; FABP-4, was significantly increased in the PC (*p* < 0.001) and EPJ 0.01 mg/mL groups compared to the C (*p* < 0.01). Conversely, PPARγ and FABP-4 mRNA expression in the EPJ 0.02 mg/mL group was not significantly different from the C group. In the EPJ 0.02 mg/mL group, UCP-1 (*p* = 0.059), PPARα (*p* = 0.067), and ACO (*p* = 0.062) were increased compared with the C group. However, UCP-2, UCP-3 and CPT-1 were not statistically different by both EPJ groups. In conclusion, EPJ not only inhibited the expression of genes related to adipocyte differentiation and lipid accumulation but also increased expression of genes associated with thermogenesis. The EPJ 0.02 mg/mL group showed relatively potent activity compared to the EPJ 0.01 mg/mL group.

### 3.6. Characterization of Phenolic Acids and Flavonoids in EPJ

#### 3.6.1. Characterization of Phenolic Acids

**Figure 6:**
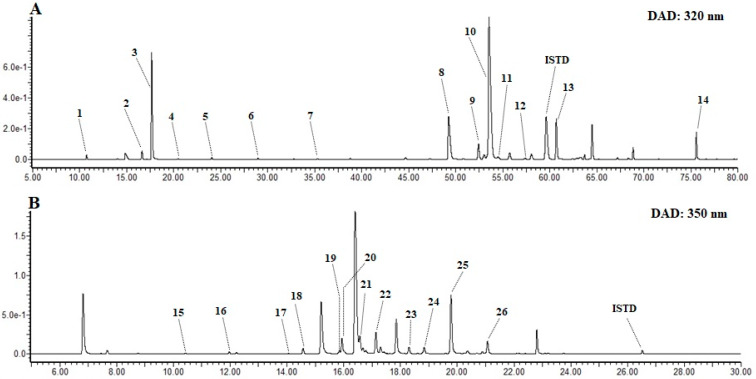
Ultra performance liquid chromatography coupled with diode array detector (UPLC-DAD) chromatograms of EPJ. (**A**) Phenolic acids at 320 nm, and (**B**) flavonoids at 350 nm. Compound names of each peak are presented in Table 1.

EPJ was characterized by UPLC-DAD-QToF-MS for polyphenolic compounds. Compound identification was done using a UV-absorbance pattern, mass spectral data under (+) ESI mode, literature data, and by comparing with an authentic standard, wherever available. A total of fourteen phenolic acids and twelve flavonols, which were identified from EPJ, are presented in Table 1 and Figure 6. 

Peak **2** showed the base peak at *m/z* 163 corresponding to [Caf+H-H_2_O]^+^ and minor fragment ions at *m/z* 181[Caf+H]^+^, 145[Caf+H-2H_2_O]^+^ and 135[Caf+H-H_2_O-CO]^+^. Comparing these fragments and retention time with the authentic standard, peak **2** was identified as caffeic acid. Peak **1**, **3**, **4**, and **5** yielded parent ion ([M+H]^+^) at *m/z* 355. Adduct ions at *m/z* 377 [M+Na]^+^ and 393 [M+K]^+^ were also observed. The base peak at *m/z* 163 corresponding to [Caf+H-H_2_O]^+^, 100% relative abundance (RA) revealed these compounds are caffeoyl derivatives. Other caffeic acid related fragment ions were also observed at lower RA. Peak **1**, **3,** and **4** further compared with the authentic standards and identified as 3-*O*-caffeoyl quinic acid (3CQA), 5CQA and 4CQA, respectively. Based on the elution order CQA isomers in reverse phase chromatographic column, peak **5** tentatively identified as *cis*-5CQA [31]. Peak **7** showed 14 Da increment in its [M+H]^+^, [M+Na]^+^ and [M+K]^+^ ions compared to CQAs. Base peak at *m/z* 163 (100% RA) and fragment ions related to caffeoyl moieties were produced. Comparing it with authentic standard peak **7** was identified as 5-*O*-caffeoylquinic acid methyl ester (5CQM). Peak **6** was assigned as feruloyl derivative from the base peak at *m/z* 177 [Fr+H-H_2_O]^+^ (100% RA), ferulic acid related fragment ions were also observed at *m/z* 195[Fr+H]^+^, 149[Caf+H-H_2_O-CO]^+^, 145[Caf+H-H_2_O-CH_3_OH]^+^ and 134[Caf+H-H_2_O-CO-CH_3_]^+^ [32]. This peak retention time further compared with the authentic standard and identified as 5-*O*-feruloylquinic acid (5FQA). Peak **8** provided fragment ions corresponding to caffeoyl moieties (*m/z* 181, 163, 145, and 135). The base peak at *m/z* 417 [M+H-H_2_O]^+^ showed 100% RA. By comparing the fragment ions and UV absorbance with literature, peak **8** was determined as fukinolic acid [29]. Single ion monitoring of total ion chromatogram at *m/z* 517 showed five peaks (**9**, **10**, **11**, **12**, and **13**). These isomers have one additional caffeic acid compared to CQA and provided a base peak at *m/z* 499[M+H-Caf-H_2_O]^+^ (100% RA). Assignment of peaks was done by comparing with authentic standards and literature data, and identified as 3,4-*O*-di-caffeoylquinic acid (3,4-diCQA), 3,5-diCQA, 1,5-diCQA, *cis*-3,5-diCQA, and 4,5-diCQA, respectively [33]. Peak **14** detected at a retention time of 75.59 min showed the parent ion at *m/z* 679 and a fragment ion at *m/z* 661 [M+H-H_2_O]^+^. Base peaks at *m/z* 499 (100% RA) and 163[Caf+H-H_2_O]^+^ (75% RA) were predominant. This peak further compared with the authentic standard and identified as 3,4,5-*O*-tricaffeoylquinic acid (3,4,5-triCQA). Peaks **5**, **7**, **11**, **12**, and **14** are reported for the first time in EPJ. 

#### 3.6.2. Characterization of Flavonoids 

Twelve flavonols (peaks **15**–**26**) were identified from EPJ. Peak **15** produced a parent ion at *m/z* 669, minor fragment ion of [M+H-Ac-Glu]^+^ at *m/z* 465, and *m/z* 303 corresponds to quercetin aglycone; hence, this compound identified as quercetin 3-*O*-(6″-*O*-acetyl) glucoside-7-*O*-glucoside. Peak **16** yielded [M+H]^+^ ion at *m/z* 653 (100% RA) and fragment ions at *m/z* 449 after the removal of acetylated glucose moiety ([M+H-Ac-Glu]^+^) so, it was assigned as kaempferol 3-*O*-(6″-*O*-acetyl) glucoside-7-*O*-glucoside. Peaks **17**, **18**, and **19** showed quercetin aglycone. The fragment ion at *m/z* 465 [M+H-Rham]^+^, and 303 [M+H-Rut]^+^ suggested that there is a rutinoside conjugate on peak **17** while, only one sugar unit was detected on peak **18** (*m/z* 303 [M+H-Glu]^+^). Peak **19** produced a fragment ion at *m/z* 303 due to the removal of [M+H-Mal-Glu]^+^. Hence, by comparing with the literature data, these peaks were identified as quercetin 3-*O*-rutinoside (rutin), quercetin 3-*O*-glucoside (isoquercitrin), and quercetin 3-*O*-(6″-*O*-malonyl) glucoside, respectively [34]. Peaks **20**, **21**, and **23** showed similar fragmentation patterns (Table 1) with peaks **17**, **18**, and **19,** except the aglycon ion at *m/z* 287, which corresponds to kaempeferol aglycone. Therefore, these peaks assigned as kaempferol 3-*O*-rutinoside (nicotiflorin), kaempferol 3-*O*-glucoside (astragalin), and kaempferol 3-*O*-(6″-*O*-malonyl) glucoside, respectively. Peaks **22** and **23** produced the [M+H-Ac-Glu]^+^ fragment ion at *m/z* 303 and 287, and assigned as quercetin 3-*O*-(6″-*O*-acetyl) glucoside and kaempferol 3-*O*-(6″-*O*-acetyl) glucoside, respectively [29,35]. Finally, peaks **24** and **26** yielded a fragment ion related to caffeoyl moieties (*m/z* 181, 163, 145, and 135). Moreover, [M+H-Caf-Glu]^+^ fragment ions at *m/z* 303 and 287 revealed that these peaks are caffeoyl glucoside conjugate; therefore, they were identified as quercetin 3-*O*-(6″-*O*-caffeoyl) glucoside and kaempferol 3-*O*-(6″-*O*-caffeoyl) glucoside, respectively. This is the first report on the presence of peaks **15**, **16**, **17**, **19**, **20**, **23**, and **24** in EPJ.

Quantification of polyphenolic compounds was conducted from peak areas of the (UPLC-DAD) chromatogram at 320 and 350 nm for phenolic acid and flavonoids, respectively, using internal standards (Table 1). The total phenolic acid content was 1676.4 ± 42.0 mg/ 100 g DW, with 3,5diQA (870.1 ± 14.5 mg/ 100 g DW) as the principal compound followed by 5CQA (299.5 ± 20.3 mg/ 100 g DW), and fukinolic acid (235.0 ± 4.8 mg/ 100 g DW). Of total flavonoid, 1064.8 ± 124.6 mg/ 100 g DW was determined in EPJ. Kaempferol 3-*O*-(6″-*O*-acetyl) glucoside (429.6 ± 51.4 mg/ 100 g DW) was presented in a higher amount. The contents of quercetin 3-*O*-(6″-*O*-acetyl) glucoside (140.9 ± 16.4 mg/ 100 g DW), astragalin (132.6 ± 15.7 mg/ 100 g DW), nicotiflorin (112.1 ± 13.2 mg/ 100 g DW) were also significant compared to the other flavonoids identified.

## 4. Discussion

In this study, the cytokine release of macrophages and adipocytes were evaluated in vitro to investigate the inhibitory effects of EPJ on obesity-related inflammatory responses. The study also evaluated its anti-adipogenic effect of EPJ by measuring the inhibition of adipocyte differentiation and lipid accumulation related mRNA expression level. The results showed that EPJ reduced NO secretion (Figure 1) in addition to the protein and mRNA expression levels of MCP-1 and TNFα in LPS-induced RAW 264.7 macrophages (Figure 2) in a dose dependent manner. In the differentiated 3T3-L1 adipocytes, the EPJ showed adipocytokine improvements such as MCP-1 and adiponectin (Figure 3) and anti-lipogenic effect (Figure 4). 

The LPS-stimulated NO production in RAW 264.7 was reduced in the EPJ 0.1 mg/mL group without cell toxicity (Figure 1). NO is biosynthesized endogenously by various NOS enzymes [36]. Previous studies have shown that anti-allergic and anti-inflammatory effects of Bakkenolide B isolated from PJ leaves were found to inhibit the production of LPS-induced nitric oxide synthase (NOS) and cyclooxygenase-2 (COX-2) protein within peritoneal macrophage cells [22]. Despite not a single compound (i.e., Bakkenolide B), the results of this study showed the inhibitory effect of EPJ on NO production. 

In the inflammatory condition, TNFα induces chemokines such as interleukin-8 (IL-8) and MCP-1 to mediate the initial inflammatory responses [37]. A study on EPJ reported that it reduces elevated levels of cytokines, including TNFα in the Jurkat T-cell line and decreased interleukin-6 (IL-6) in THP-1 cells (a human monocytic cell line) [23]. The current result revealed that MCP-1 and TNFα protein levels were significantly decreased in the PC and EPJ 0.1 mg/mL groups for LPS-treated RAW 264.7 macrophages, and also, the mRNA level was decreased by EPJ (Figure 2). 

As a PPARγ ligand, TZDs are known for adipocyte differentiation and have an anti-inflammatory effect [38]. Figure 5 shows the mRNA expression level of several genes involved in lipid accumulation and degradation. PPARγ is a key transcriptional factor of adipocyte differentiation, also involved in the treatment of type 2 diabetes and the improvement of metabolic syndrome. Interestingly, EPJ activated PPARγ like the TZD effect but inhibited differentiation in 3T3-L1 cells, as shown in Figure 4. Figure 4 shows the effect of EPJ on lipid accumulation in differentiated adipocytes without cell cytotoxicity, showing the possibility of the prevention of obesity due to the decrease in adipocyte accumulation of PJ. This result is attributed to the combination of EPJ, the PPARα and the activation of heat-related factor UCP-1. 

This result suggests that EPJ increased not only the expression of the PPARγ and target genes (i.e., MCP-1, TNFα, Adiponectin and UCP-1) but also the PPARα activation, which affects energy metabolism in white adipose tissue (WAT) and its target genes [39]. Furthermore, activation of PPARγ influenced adiponectin and UCP-1 increase in adipocytes as well as NO, MCP-1, and TNFα reduction in macrophages or adipocytes. However, further investigation is necessary as to whether PJ acts as an agonist of PPARα and γ through direct ligand assay.

A previous study showed that treatment with EPJ (flower buds) 3T3-L1 adipocytes significantly decreased the expression of the adipogenic transcription factors, such as sterol regulatory element-binding proteins-1c (SREBP-1c), PPARγ, and CCAAT-enhancer-binding proteins (C/EBPα) [40]. On the other hand, our results showed that the PPARγ and its target gene FABP-4 were significantly increased in the PC group treated with TZD and in the EPJ 0.01 mg/mL treated group. However, compared with the PC group, FABP-4 was significantly decreased in the two groups treated with EPJ (Figure 5A). 

UCPs (UCP-1, -2, and -3), have been identified as mediators of thermogenesis, a group of mitochondrial proton transporters responsible for uncoupling respiration from oxidative phosphorylation [41]. According to a previous study, UCP-1 expression in beige adipocytes was increased by beta adrenergic receptor activation, and 3T3-L1 cells can differentiate into beige-like adipocytes with prolonged treatment of T3, IBMX, and rosiglitazone [42]. TZD treatment of db/db mice also induced UCP-1 and UCP-3 expression in brown adipose tissue [43]. In Figure 5A, PPARγ mRNA expression levels were increased in the PC and EPJ 0.01 mg/mL groups, indicating that EPJ also has PPARγ activation effects similar to TZD as a PPARγ agonist. The results indicated the increase of UCP-1 mRNA levels in the PC and EPJ 0.02 mg/mL groups by PPARγ activation (Figure 5B). In differentiated 3T3-L1 cells treated with EPJ 0.02 mg/mL, PPARα, ACO, and CPT-1 genes increased compared with the C group (Figure 5C). The target genes of PPARα at the cellular level are involved in the catabolism of lipids [44], and ACO and CPT-1 have been extensively linked to fatty acid oxidation [45].

The available evidence revealed that dietary polyphenols have many health benefits ranging from infectious disease to chronic metabolic syndromes and cancers. The chemical constituents presented in PJ have shown anti-allergic properties [46], anti-inflammatory [35], radical scavenging activities [35], and macrophage NO production inhibiting activities [47]. These activities are related with fukinolic acid, fukiic acid, and other hydroxycinnamoylquinic acid derivatives identified from the methanol extract of PJ [48]. The current biological activities observed can be justified strongly due to the presence of higher concentration of flavonoids and phenolic acids in the ethanol extract of PJ. 

In conclusion, EPJ treatment decreased the inflammatory response and lipid accumulation but increased anti-inflammatory and thermogenesis response in macrophages and adipocytes. These results suggest that the consumption of PJ can contribute to the prevention and improvement of obesity and related inflammatory conditions. Further research is required about PJ and their mechanisms in obesity and the related inflammation. We are conducting animal experiments to reproduce the positive results shown in vitro and to determine the mechanism of PJ. The results of this experiment are expected to further support the health benefits of PJ as a Korean agrofood.

## Figures and Tables

**Table 1:** Ultra performance liquid chromatography coupled with diode array detector, quadrupole time-of-flight mass spectrometry (UPLC-QToF-MS) characterization and contents of polyphenolic compounds from EPJ.

Peak no.	Compound Name	RT (min)	DAD λmax (nm)	Formula [M+H]^+^	ESI(+)-QToF/MS (Experimental Ions, *m/z*)		Content (mg/ 100 g DW)
					Exp. Mass [M+H]^+^	Adducts and Fragmentation (*m/z*)	
Phenolic acid							
**2 ^b^**	caffeic acid	16.63	240_sh_,295_sh_,323	C_9_H_9_O_4_	181.0727	163[M+H-H_2_O]^+^, 145[M+H-2H_2_O]^+^, 135[M+H-H_2_O-CO]^+^	24.4 ± 0.3
**1** ^b^	3-*O*-caffeoylquinic acid (neochlorogenic acid)	10.73	240_sh_,295_sh_,324	C_16_H_19_O_9_	355.0504	393[M+K]^+^, 377[M+Na]^+^, 337[M+H-H_2_O]^+^, 181[Caf+H]^+^, 163[Caf+H-H_2_O]^+^, 145[Caf+H-2H_2_O]^+^, 135[Caf+H-H_2_O-CO]^+^	10.5 ± 0.2
**3** ^b^	5-*O*-caffeoylquinic acid (chlorogenic acid)	17.65	242_sh_,299_sh_,325	C_16_H_19_O_9_	355.0504	393[M+K]^+^, 377[M+Na]^+^, 337[M+H-H_2_O]^+^, 181[Caf+H]^+^, 163[Caf+H-H_2_O]^+^, 145[Caf+H-2H_2_O]^+^, 135[Caf+H-H_2_O-CO]^+^	299.5 ± 20.3
**4** ^b^	4-*O*-caffeoylquinic acid (cryptochlorogenic acid)	20.48	241_sh_,299_sh_,324	C_16_H_19_O_9_	355.0504	393[M+K]^+^, 377[M+Na]^+^, 337[M+H-H_2_O]^+^, 181[Caf+H]^+^, 163[Caf+H-H_2_O]^+^, 145[Caf+H-2H_2_O]^+^, 135 [Caf+H-H2O-CO]^+^	2.1 ± 0.1
**5** ^a^	*cis*-5-*O*-caffeoylquinic acid (*cis*-chlorogenic acid)	24.04	232,314	C_16_H_19_O_9_	355.0504	393[M+K]^+^, 377[M+Na]^+^, 337[M+H-H_2_O]^+^, 181[Caf+H]^+^, 163[Caf+H-H_2_O]^+^, 145[Caf+H-2H_2_O]^+^, 135[Caf+H-H_2_O-CO]^+^	6.1 ± 0.1
**6** ^b^	5-*O*-feruloylquinic acid	28.96	234,296_sh_,325	C_17_H_21_O_9_	369.0629	407[M+K]^+^, 391[M+Na]^+^, 351[M+H-H_2_O]^+^, 195[Fr+H]^+^, 177[Fr+H-H_2_O]^+^, 149[Fr+H-H_2_O-CO]^+^, 145[Fr+H-H_2_O-CH_3_OH]^+^, 134[Fr+H-H_2_O-CO-CH_3_]^+^	3.8 ± 0.1
**7** ^a,b^	5-*O*-caffeoylquinic acid methyl ester	35.28	244 _sh_,296_sh_,326	C_17_H_21_O_9_	369.0629	407[M+K]^+^, 391[M+Na]^+^, 351[M+H-H_2_O]^+^, 181[Caf+H]^+^, 163[Caf+H-H_2_O]^+^, 145[Caf+H-2H_2_O ]^+^, 135[Caf+H-H2O-CO]^+^	3.2 ± 0.2
**8**	fukinolic acid	49.23	226 _sh_,288_sh_,329	C_20_H_19_O_11_	435.0651	473[M+K]^+^, 457[M+Na]^+^, 417[M+H-H_2_O]^+^, 181[Caf+H]^+^, 163[Caf+H- H_2_O ]^+^, 145[Caf+H-2H_2_O ]^+^, 135[Caf+H-H_2_O-CO]^+^	235.0 ± 4.8
**9** ^b^	3,4-di-*O*-caffeoylquinic acid (isochlorogenic acid B)	52.42	241_sh_,297_sh_,324	C_25_H_25_O_12_	517.0995	555[M+K]^+^, 539[M+Na]^+^, 499[M+H-H_2_O]^+^, 355[M+H-Caf]^+^, 337[M+H-Caf-H_2_O]^+^, 181[Caf+H]^+^, 163[Caf+H-H_2_O]^+^, 145[Caf+H-2H_2_O]^+^, 135[Caf+H-H_2_O-CO]^+^	60.5 ± 1.5
**10** ^b^	3,5-di-*O*-caffeoylquinic acid (isochlorogenic acid A)	53.53	241_sh_,299_sh_,326	C_25_H_25_O_12_	517.0995	555[M+K]^+^, 539[M+Na]^+^, 499[M+H-H_2_O]^+^, 355[M+H-Caf]^+^, 337[M+H-Caf-H_2_O]^+^, 181[Caf+H]^+^, 163[Caf+H-H_2_O]^+^, 145[Caf+H-2H_2_O]^+^, 135[Caf+H-H_2_O-CO]^+^	870.1 ± 14.5
**11** ^a,b^	1,5-di-*O*-caffeoylquinic acid (isochlorogenic acid A)	54.49	241_sh_,298_sh_,325	C_25_H_25_O_12_	517.0995	555[M+K]^+^, 539[M+Na]^+^, 499[M+H-H_2_O]^+^, 355[M+H-Caf]^+^, 337[M+H-Caf-H_2_O]^+^, 181[Caf+H]^+^, 163[Caf+H-H_2_O]^+^, 145[Caf+H-2H_2_O]^+^, 135[Caf+H-H_2_O-CO]^+^	12.2 ± 0.3
**12** ^a^	3-*O*-*cis*-caffeoyl-5-*O*-caffeoylquinic acid (*cis*-isochlorogenic acid A)	57.38	243_sh_,296_sh_,316	C_25_H_25_O_12_	517.0995	555[M+K]^+^, 539[M+Na]^+^, 499[M+H-H_2_O]^+^, 355[M+H-Caf]^+^, 337[M+H-Caf-H_2_O]^+^, 181[Caf+H]^+^, 163[Caf+H-H_2_O]^+^, 145[Caf+H-2H_2_O]^+^, 135[Caf+H-H_2_O-CO]^+^	5.4 ± 0.4
**13** ^b^	4,5-di-*O*-caffeoylquinic acid (isochlorogenic acid C)	60.69	242_sh_,299_sh_,326	C_25_H_25_O_12_	517.0995	555[M+K]^+^, 539[M+Na]^+^, 499[M+H-H_2_O]^+^, 355[M+H-Caf]^+^, 337[M+H-Caf-H_2_O]^+^, 181[Caf+H]^+^, 163[Caf+H-H_2_O]^+^, 145[Caf+H-2H_2_O]^+^, 135[Caf+H-H_2_O-CO]^+^	77.8 ± 1.3
**14** ^a,b^	3,4,5-di-*O*-caffeoylquinic acid	75.59	243,297_sh_,326	C_34_H_31_O_15_	679.1178	717[M+K]^+^, 701[M+Na]^+^, 661[M+H-H_2_O]^+^, 517[M+H-Caf]^+^, 499[M+H-Caf-H_2_O]^+^, 337[M+H-2Caf-H_2_O]^+^, 181[Caf+H]^+^, 163[Caf+H-H_2_O]^+^, 145[Caf+H-2H_2_O]^+^, 135[Caf+H-H_2_O-CO]^+^	65.8 ± 0.4
Total Phenolic acid content							1676.4 ± 42.0
Flavonoids							
**15** ^a^	quercetin 3-*O*-(6″-*O*-acetyl) glucoside-7-*O*-glucoside	10.11	255,268_sh_, 301_sh_ 345	C_29_H_33_O_18_	669.1531	691[M+Na] ^+^, 465[M+H-Ac-Glu] ^+^, 303[M+H-Ac-2Glu] ^+^	1.0 ± 0.1
**16** ^a^	kaempferol 3-*O*-(6″-*O*-acetyl) glucoside-7-*O*-glucoside	11.97	263,331	C_29_H_33_O_17_	653.1549	675[M+Na]^+^, 491[M+H-Glu]^+^, 449[M+H-Ac-Glu]^+^, 287[M+H-Ac-2Glu]^+^	11.2 ± 1.2
**17** ^a^	quercetin 3-*O*-rutinoside (rutin)	14.06	257,265_sh_, 297_sh_, 354	C_27_H_31_O_16_	611.1428	633[M+Na] ^+^, 465[M+H-Rham] ^+^, 303[M+H-Rut] ^+^	3.6 ± 0.5
**18**	quercetin 3-*O*-glucoside (isoquercitrin)	14.57	257,265_sh_, 298_sh_,356	C_21_H_21_O_12_	465.0908	487[M+Na] ^+^, 303[M+H-Glu] ^+^	36.3 ± 4.2
**19** ^a^	quercetin 3-*O*-(6″-*O*-malonyl) glucoside	15.83	238,291_sh_, 335	C_24_H_23_O_15_	551.0884	573[M+Na] ^+^, 303[M+H-Mal-Glu] ^+^	18.9 ± 2.2
**20** ^a^	kaempferol 3-*O*-rutinoside (nicotiflorin)	15.94	266,347	C_27_H_31_O_15_	595.1497	617[M+Na] ^+^, 449[M+H-Rham] ^+^, 287[M+H-Rut] ^+^	112.1 ± 13.2
**21**	kaempferol 3-*O*-glucoside (astragalin)	16.57	269,342	C_21_H_21_O_11_	449.0971	471[M+Na]^+^, 287[M+H-Glu] ^+^	132.6 ± 15.7
**22**	quercetin 3-*O*-(6″-*O*-acetyl) glucoside	17.13	257,266_sh_,296_sh_, 355	C_23_H_23_O_13_	507.1000	529[M+Na] ^+^, 303[M+H-Ac-Glu] ^+^	140.9 ± 16.4
**23** ^a^	kaempferol 3-*O*-(6″-*O*-malonyl) glucoside	18.30	266,298_sh_,351	C_27_H_31_O_15_	535.0933	557[M+Na] ^+^, 287[M+H-Mal-Glu] ^+^	44.9 ± 5.3
**24** ^a^	quercetin 3-*O*-(6″-*O*-caffeoyl) glucoside	18.83	254,267_sh_,301_sh_, 334	C_30_H_27_O_15_	627.1175	649[M+Na] ^+^, 303[M+H-Caf-Glu] ^+^, 181[Caf+H] ^+^, 163[Caf+H-H_2_O] ^+^, 145[Caf+H-2H_2_O] ^+^, 135[Caf+H-H_2_O-CO] ^+^	50.8 ± 6.1
**25**	kaempferol 3-*O*-(6″-*O*-acetyl) glucoside	19.78	266,300_sh_,348	C_23_H_23_O_12_	491.1042	513[M+Na] ^+^, 287[M+H-Ac-Glu] ^+^	429.6 ± 51.4
**26** ^a^	kaempferol 3-*O*-(6″-*O*-caffeoyl) glucoside	21.07	267,330	C_27_H_31_O_14_	611.1226	633[M+Na]^+^, 287[M+H-Caf-Glu]^+^, 181[Caf+H]^+^, 163[Caf+H-H_2_O] ^+^, 145[Caf+H-2H_2_O] ^+^, 135[Caf+H- H_2_O-CO] ^+^	82.9 ± 10.8
Total flavonoid content							1064.8 ± 124.6

All samples analyzed in positive ESI-ionization mode (*m/z*, [M+H]^+^) of QToF/MS; [M+K]^+^, [M+Na]^+^, and adducts presented; Caf: caffeic acid (180 Da) or caffeoyl (162 Da); Fr: ferulic acid (194 Da) or feruloyl (176 Da); Glu: glucose (162 Da); Rham: rhaminose (146 Da); Rut: rutinose (308 Da); Ac: acetyl (42 Da); peak assignment was done by comparing UV-visible, MS fragmentation spectra and authentic standards, wherever available; (^a^) new compound identified; (^b^) further confirmed in comparison with authentic standards.
